# Chrysin Ameliorates Malfunction of Retinoid Visual Cycle through Blocking Activation of AGE-RAGE-ER Stress in Glucose-Stimulated Retinal Pigment Epithelial Cells and Diabetic Eyes

**DOI:** 10.3390/nu10081046

**Published:** 2018-08-08

**Authors:** Min-Kyung Kang, Eun-Jung Lee, Yun-Ho Kim, Dong Yeon Kim, Hyeongjoo Oh, Soo-Il Kim, Young-Hee Kang

**Affiliations:** Department of Food Science and Nutrition, Hallym University, Chuncheon 24252, Korea; mitoly@hallym.ac.kr (M.-K.K.); reydmswjd@naver.com (E.-J.L.); royalskim@hallym.ac.kr (Y.-H.K.); ehddus3290@naver.com (D.Y.K.); ohhyeongju@gmail.com (H.O.); ky4850@naver.com (S.-I.K.)

**Keywords:** advanced glycation end products, chyrsin, diabetic retinopathy, endoplasmic reticulum, retinal pigment epithelium, visual cycle

## Abstract

Diabetes-associated visual cycle impairment has been implicated in diabetic retinopathy, and chronic hyperglycemia causes detrimental effects on visual function. Chrysin, a naturally occurring flavonoid found in various herbs, has anti-inflammatory, antioxidant, and neuroprotective properties. The goal of the current study was to identify the retinoprotective role of chrysin in maintaining robust retinoid visual cycle-related components. The in vitro study employed human retinal pigment epithelial (RPE) cells exposed to 33 mM of glucose or advanced glycation end products (AGEs) in the presence of 1–20 μM chrysin for three days. In the in vivo study, 10 mg/kg of chrysin was orally administrated to db/db mice. Treating chrysin reversed the glucose-induced production of vascular endothelial growth factor, insulin-like growth factor-1, and pigment epithelium-derived factor (PEDF) in RPE cells. The outer nuclear layer thickness of chrysin-exposed retina was enhanced. The oral gavage of chrysin augmented the levels of the visual cycle enzymes of RPE65, lecithin retinol acyltransferase (LRAT), retinol dehydrogenase 5 (RDH5), and rhodopsin diminished in db/db mouse retina. The diabetic tissue levels of the retinoid binding proteins and the receptor of the cellular retinol-binding protein, cellular retinaldehyde-binding protein-1, interphotoreceptor retinoid-binding protein and stimulated by retinoic acid 6 were restored to those of normal mouse retina. The presence of chrysin demoted AGE secretion and AGE receptor (RAGE) induction in glucose-exposed RPE cells and diabetic eyes. Chrysin inhibited the reduction of PEDF, RPE 65, LRAT, and RDH5 in 100 μg/mL of AGE-bovine serum albumin-exposed RPE cells. The treatment of RPE cells with chrysin reduced the activation of endoplasmic reticulum (ER) stress. Chrysin inhibited the impairment of the retinoid visual cycle through blocking ER stress via the AGE-RAGE activation in glucose-stimulated RPE cells and diabetic eyes. This is the first study demonstrating the protective effects of chrysin on the diabetes-associated malfunctioned visual cycle.

## 1. Introduction

Diabetic retinopathy (DR) is a microvascular eye disease involving retinal neurodegeneration [1]. DR is associated strongly with a prolonged duration of hyperglycemia and hypertension, in which serious damage occurs to the retina, consequently causing vision loss and blindness [2,3]. In the early stage of DR, hyperglycemia can cause blood vessels in the retina to leak fluid, making retina and macula swell [1,4]. In the advanced stage of DR, abnormal new blood vessels can outgrow on the surface of the retina, and scar tissues and tiny exudate particles can form in the retina [1,3,5]. Here, new vessels are fragile, and occasionally bleed into the vitreous, which is called vitreous hemorrhage [4]. These abnormal alterations in the macula and retina can steal central and peripheral vision [2,3,4]. Pathophysiological factors germane to the development of DR include genetic and epigenetic factors, free radicals, advanced glycosylation end products (AGEs), and inflammatory factors [1]. Increasing evidence highlights inflammation in regard to the induction of diabetes-mediated biochemical and molecular alterations in the retina, ultimately contributing to retinal complications and vision loss [1,4]. However, the molecular mechanisms underlying inflammatory pathways are not concretely defined in DR. On the other hand, the aberrant functions in the mitochondria and endoplasmic reticulum (ER) are recognized as key players promoting the apoptotic demise of retinal vascular and neuronal cells in diabetic eyes [6,7]. The apoptotic death of retinal cells such as photoreceptors, neurons, and vascular cells directly affects visual function in DR and retinitis pigmentosa [7,8].

Several studies have addressed the putative role of ER stress in the visual system [9,10,11]. The retinal pigment epithelial (RPE) cells support the light-sensitive photoreceptor cells in the retina, which are crucial for visual function [12]. The ER stress in these cells promotes RPE injury in DR [13,14]. Inhibition of aldose reductase alleviates hyperglycemia-induced RPE cell death and ER stress, and prevents retinal degeneration in the diabetic eye [15]. In addition, methylglyoxal, the AGE adduct in DR, reduces RPE cell viability via the ER stress-dependent intracellular reactive oxygen species (ROS) production, mitochondrial membrane potential loss, and intracellular calcium increase [16]. Thus, reducing or blocking ER stress may be a therapeutic option for preventing DR. The retinoid visual cycle spanning photoreceptor cells and the underlying RPE is the cyclical processing of retinol by which 11-cis-retinal is regenerated from all-trans-retinal, following a photoisomerization event entailing enzymes broadly classified as acyltransferases, short-chain dehydrogenases/reductases, and carotenoid/retinoid isomerases/oxygenases [12,17,18]. Several animal models determine the mechanisms that underlie RPE65-associated retinal dystrophies [17]. Recessive blinding diseases are attributed to mutations or deficiency in RPE65, a key isomerase converting light-insensitive all-trans-retinyl ester to light-sensitive 11-cis-retinol for continued visual function [17,18,19].

**Figure 1:**
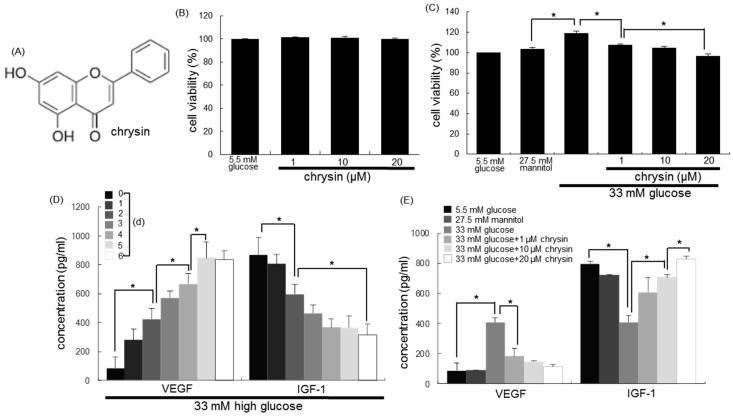
Chemical structure (**A**) and retinal pigment epithelial cytotoxicity (**B**) of chrysin, and the inhibitory effects of chrysin on cell proliferation by glucose (**C**), and temporal responses (**D**) and the inhibition of antivascular endothelial growth factor (VEGF) and insulin-like growth factor-1 (IGF-1) secretion by chrysin (**E**) in human retinal pigment epithelial (RPE) cells. Cells were incubated with 33 mM of glucose in the absence and presence of between 1–20 μM chrysin for up to six days. Cells were also incubated with 5.5 mM of glucose and 27.5 mM of mannitol as osmotic controls. After RPE cells were cultured in high glucose media, cell viability was measured by using MTT (3-(4,5-dimethylthiazol-2-yl)-2,5-diphenyltertrazolium bromide) assay (**B** and **C**, 100% viability with 5.5 mM of glucose). The secretion of VEGF and IGF-1 was measured with commercial ELISA kits (**D** and **E**). * Values in bar graphs (mean ± standard error of the mean (SEM), *n* = nine independent experiments) indicate significant different at *p* < 0.05.

Numerous pharmacologic agents such as antivascular endothelial growth factor (VEGF), aldose reductase inhibitors, and protein kinase C inhibitors have been suggested as therapeutic potential ones for ocular diseases [20,21,22]. However, these inhibitors have failed to demonstrate significant efficacy in the treatment of DR in clinical trials. Natural products have been developed as agents with minimal side effects for intraocular proliferation and angiogenesis [23,24]. Evidence is emerging that lutein and zeaxanthin protect against visual disorders, including age-related macular degeneration, retinitis pigmentosa, and DR [25,26]. The mechanism(s) underscoring the prevention of eye diseases by hydroxycarotenoids may come from their antioxidant, neuroprotective, and anti-inflammatory functions in the retina [26]. Chrysin (Figure 1A) is a flavone-type flavonoid that is present in honey, propolis, honeycomb, and passion flowers, and exhibits multiple biological effects, including anti-inflammation and neuroprotection [27,28]. One safety study reports that the recommended daily concentrations of chrysin are 0.5 g to 3 g [29]. Following intake by humans, chrysin has low oral bioavailability and rapid fecal elimination, in which its major form in plasma is chrysin sulphate [28,30]. Our previous study showed that the multifunctional chrysin exerted the retinoprotection through inhibiting diabetes-associated retinal neovascularization and blood–retinal barrier breakdown [31]. However, whether chrysin is capable of preventing vision loss in diabetic ocular diseases remains unclear. The current study attempted to investigate that chrysin ameliorated glucose-induced visual damage in RPE cells and in mouse diabetic models. This study examined the mechanisms germane to visual cycle dysfunction in RPE, and the contribution of AGE-RAGE system and ER stress to the visual impairment in DR.

## 2. Materials and Methods

### 2.1. Materials

Fetal bovine serum (FBS), trypsin-ethylenediaminetetraacetic acid (EDTA), and penicillin–streptomycin were supplied by Lonza (Walkersvillle, MD, USA). Dulbecco’s modified eagle medium (DMEM, low glucose) media, mannitol, D-glucose, tunicamycin, and thapsigargin were obtained from Sigma-Aldrich Chemical (St. Louis, MO, USA), as were all other reagents, unless specifically stated elsewhere. Mouse monoclonal antibodies of pigment epithelium-derived factor (PEDF, Lot# E2813), RPE65 (Lot# I2816), interphotoreceptor retinoid-binding protein (IRBP, Lot# A1315), and receptor for advanced glycation end product (RAGE, Lot# J2616) were provided from Santa Cruz Biotechnology (Santa Cruz, CA, USA). Mouse monoclonal antibodies of rhodopsin and cellular retinaldehyde-binding protein-1 (CRALBP, Lot# ab15015), rabbit monoclonal retinol dehydrogenase 5 (RDH5, Lot# ab200197) antibody, and rabbit polyclonal antibodies of activating transcription factor 6 (ATF6, Lot# ab37149) and 78 kDa glucose-regulated protein/binding immunoglobulin protein (GRP78/Bip, Lot# ab53068) were obtained by Abcam Biochemicals (Cambridge, UK). Rabbit polyclonal antibodies of advanced glycation end product (AGEs, Lot# AG01111024) and stimulated by retinoic acid 6 (STRA6, Lot# BS12351R) were purchased from Bioss (Boston, MA, USA). Rabbit polyclonal cellular retinol-binding protein (CRBP, Lot # NBP2-20132) antibody was supplied from Novus Biologicals (Littleton, CO, USA). Rabbit polyclonal inositol-requiring enzyme 1α (IRE1α) antibody (Lot# 3294S) was provided from cell signaling (Danvers, MA, USA), and rabbit polyclonal lecithin retinol acyltransferase (LRAT, Lot# MBS8508176) antibody was purchased from Mybiosource (San Diego, CA, USA). All of the antibodies for Western blot analysis were diluted at 1:1000 ratio, according to the manufacture’s instruction. Horseradish peroxidase (HRP)-conjugated goat anti-rabbit immunoglobulin (Ig)G and goat anti-mouse were purchased from Jackson ImmumnoReserch Laboratories (West Grove, PA, USA). Advanced glycation end product-bovine serum albumin (AGE-BSA) was provided by Merck Millipore (Billerica, MA, USA).

Chrysin (Sigma-Aldrich Chemical) was dissolved in dimethyl sulfoxide (DMSO) for live culture with cells; a final culture concentration of DMSO was <0.5%.

### 2.2. RPE Cell Culture

Primary human RPE cells were obtained from Lonza (Walkersvillle, MD, USA). Cells were grown in DMEM containing 2% FBS, 100 U/mL of penicillin, 100 μg/mL of streptomycin, 2 mM of glutamine, and 1 μg/mL of human fibroblast growth factor basic at 37 °C humidified atmosphere of 5% CO_2_ in air. RPE cells were subcultured at 90% confluence and used for further experiments within 10 passages. To induce hyperglycemia, RPE cells were incubated in media containing normal glucose (5.5 mM of d-glucose), 27.5 mM of mannitol (+5.5 mM d-glucose) as an osmotic control, or high glucose (33 mM of d-glucose) in the absence and presence of 1–20 μM chrysin for up to 72 h.

The RPE cell viability was determined by assaying with MTT (3-(4,5-dimethylthiazol-2-yl)-2,5-diphenyltertrazolium bromide). RPE cells seeded at a density of 1×10^4^ cells/mL on a 24-well plate were treated with 1–20 μM chrysin in different glucose media. Cells were incubated with 1 mg/mL of MTT solution at 37 °C for 3 h, forming an insoluble purple formazan product that was dissolved in 250 µL of isopropanol. Optical density was measured using a microplate reader at the wavelength of 570 nm. Chrysin at the doses of 1–20 μM had no cytotoxicity (Figure 1B). Thus, the current experiments employed chrysin in the range of 1–20 μM.

### 2.3. In Vivo Animal Experiments

Adult male db/db mice (C57BLKS/+Leprdb Iar; Jackson Laboratory, Sacramento, CA, USA) and their age-matched non-diabetic db/m littermates (C57BLKS/J; Jackson Laboratory) were employed in the present study. Mice were supplied by the animal facility of Hallym University, kept on a 12 h light/12 h dark cycle at 23 ± 1 °C with 50 ± 10% relative humidity under specific pathogen-free conditions, and fed a standard pellet laboratory chow diet (Cargill Agri Purina, Biopia, Korea). This study was performed with seven-week-old db/db mice, because they begin to develop diabetes (hyperglycemia) at the age of seven to eight weeks. The animals were allowed to acclimatize for a week before commencing the feeding experiments. Mice were divided into three subgroups (*n* = 9 for each subgroup). Mice of the first group were non-diabetic db/m control mice, and db/db mice were divided into two subgroups. One group of db/db mice was orally administrated 10 mg/kg chrysin daily for 10 weeks via gavage. The other group of db/db mice was administrated 0.02% DMSO as the chrysin vehicle. All of the mice were sacrificed after anesthesia with ketamine/Rompun cocktail (40 mg of ketamine and 10 mg of Rompun/kg BW) at 17–18 weeks of age. No mice were dead, and no apparent signs of exhaustion were observed during the experimental period. Chrysin treatment lowered blood levels of glycated hemoglobin HbA1C (~11.5%) and fasting glucose (~580–700 mg/dL) markedly elevated in db/db mice, indicating that chrysin had a glucose-lowering effect [32]. In addition, the 24-h urine volume of diabetic mice were higher (~20-fold) than non-diabetic controls, while chrysin administration reduced the volume by ~50% [32].

All of the animal experiments were approved by the Committee on Animal Experimentation of Hallym University, and performed in compliance with the University’s Guidelines for the Care and Use of Laboratory Animals (hallym 2013–125).

### 2.4. Western Blot Analysis

Western blot analysis was conducted using whole cell lysates prepared from RPE cells at a density of 3.5 × 10^5^ cells. Mouse eye extracts were also prepared from mice that were supplemented with 10 mg/kg chrysin for 10 weeks. Whole cell lysates and mouse eye extracts were prepared in a lysis buffer containing 1 M of β-glycerophosphate, 1% of β-mercaptoethanol, 0.5 M of NaF, 0.1 M of Na_3_VO_4_, and protease inhibitor cocktail. Cell lysates and eye tissue extracts containing equal amounts of proteins and equal volumes of culture medium supernatants were electrophoresed on 6–15% SDS-PAGE and transferred onto a nitrocellulose membrane. Non-specific binding was blocked with 5% of skim milk for 3 h. The membrane was incubated overnight at 4 °C with each primary antibody of target proteins and washed in a Tris buffered saline-Tween 20 (TBS-T) for 10 min. The membrane was then incubated for 1 h with a secondary antibody of goat anti-rabbit IgG or goat anti-mouse IgG conjugated to HRP. Each target protein level was determined by using immobilon western chemiluminescent HRP substrate (Millipore Corporation, Billerica, MA, USA) and Agfa X-ray film (Agfa-Gevaert, Mortsel, Belgium). Incubation with mouse monoclonal β-actin antibody (Sigma-Aldrich Chemical) was also performed for comparative controls. 

### 2.5. Immunohistochemical Staining

For the immunohistochemical analysis, paraffin-embedded mouse eye tissue sections (5-μm thick) were employed. The sections were placed on glass slides, deparaffinated, and hydrated with xylene and graded alcohol. The sections were pre-incubated in a boiled sodium citrate buffer (10 mM of sodium citrate, 0.05% Tween 20, pH 6.0) for antigen retrieval. A specific primary antibody against RPE65 (mouse monoclonal, Santa Cruz Biotechnology, Santa Cruz, CA, USA; 1:200 dilution) and rhodopsin (mouse monoclonal, Lot# ab5417, Abcam Biochemicals, Cambridge, UK; 1:200 dilution) was incubated overnight with the tissue sections. For the measurement of RPE65 and rhodopsin expression, the tissue section was double-stained with fluorescein isothiocyanate (FITC)-conjugated anti-mouse IgG and with Cy3-conjugated anti-mouse IgG. Nuclear staining was performed with 4′,6-diamidino-2-phenylindole (DAPI, Santa Cruz Biotechnology, Santa Cruz, CA, USA). The stained tissue sections were examined using an optical Axiomager microscope system (Zeiss, Göttingen, Germany), and five images (×200) were taken for each section.

### 2.6. Enzyme-Linked Immunosorbent Assay (ELISA)

Following culture protocols, the secretion of PEDF, VEGF, and insulin-like growth factor-1 (IGF-1) in RPE cells was determined in collected culture medium supernatants by using ELISA kits (R&D Systems, Minneapolis, MN, USA), according to the manufacturer’s instructions.

### 2.7. Data Analysis

The results are presented as mean ± SEM. Statistical analyses were carried out by using the Statistical Analysis statistical software package version 6.12 (SAS Institute, Cary, NC, USA). Significance was determined by one-way analysis of variance, followed by Duncan’s multiple range test for multiple comparisons. Differences were considered significant at *p* < 0.05.

## 3. Results

### 3.1. Modulation of Production of VEGF and IGF-1 by Chrysin 

When RPE cells were incubated in media containing 33 mM of glucose for three days, the proliferation of RPE cells was observed (Figure 1C). However, the presence of nontoxic chrysin at 1–20 μM dose-dependently attenuated their proliferation. RPE cells secrete a variety of cytokines, growth factors, and extracellular matrix components, all of which contribute to retinal and choroidal neovascularization [33,34]. This study investigated that chrysin influenced the production of VEGF and IGF-1 in glucose-exposed RPE cells. The temporal production of VEGF and IGF was examined in glucose-exposed RPE cells for six days. The VEGF secretion was steadily up-regulated for six days (Figure 1D). On the contrary, the secretion of IGF-1 was continuously diminished by glucose stimulation (Figure 1D). The addition of 1–20 μM of chrysin to 33 mM of glucose-exposed RPE cells curtailed the VEGF secretion in a dose-dependent manner, while the IGF-1 production was nearly completely restored by treating ≥1 μM of chrysin to the RPE cells (Figure 1E).

### 3.2. Restoration of PEDF Production by Chrysin

**Figure 2:**
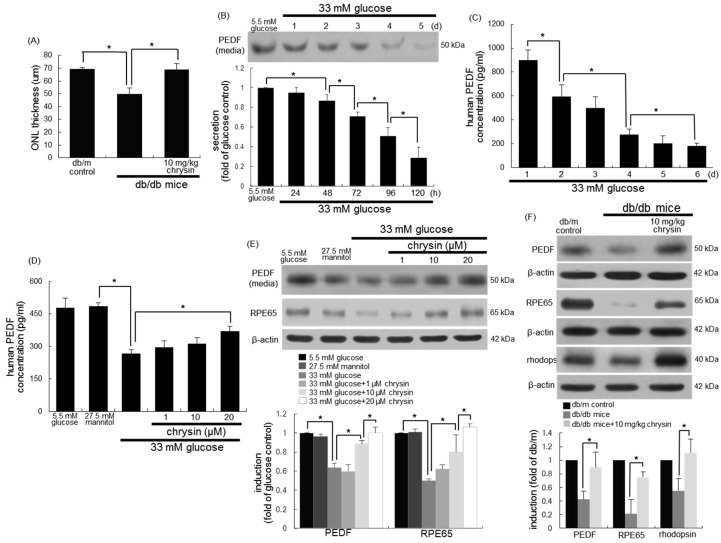
Restoration of outer nuclear layer (ONL) thickness (**A**), time course responses of production of pigment epithelium-derived factor (PEDF) by glucose (**B**,**C**) and the induction of PEDF, RPE65, and rhodopsin by chrysin (**D**–**F**). Human retinal pigment epithelial (RPE) cells were incubated with 33 of mM glucose in the absence and presence of 1–20 μM chrysin for up to 6 days. Cells were also incubated with 5.5 mM of glucose and 27.5 mM of mannitol as osmotic controls. The db/db mice were orally supplemented with 10 mg/kg of chrysin daily for 10 weeks. The db/m mice were introduced as control animals. The secretion of PEDF was measured with ELISA kits (**C**,**D**). Culture media, cell lysates, and retinal tissue extracts were subject to Western blot analysis with a primary antibody against PEDF, RPE65, and rhodopsin (**B**,**E**,**F**). β-actin protein was used as a cellular internal control for RPE cells. Bar graphs (mean ± SEM, *n* = nine independent experiments) in the bottom panels represent the densitometric results of upper blot bands. * values in bar graphs indicate significant different at *p* < 0.05.

Outer nuclear layer (ONL) thickness measured in μm was reduced in the diabetic retina (Figure 2A). This could be due to the marked loss of photoreceptors in the ONL in diabetic retina. However, the ONL thickness of the chrysin-administrated retina was enhanced. PEDF plays a clinical role in choroidal neovascularization by suppressing retinal neovascularization and endothelial cell proliferation [35]. The exposure of RPE cells to 33 mM of glucose for six days temporally reduced the PEDF secretion, as evidenced by Western blot analysis and ELISA (Figure 2B,C). When 1–20 μM chrysin was added to glucose-stimulated cells, its secretion was enhanced in a dose-dependent manner (Figure 2D,E). In addition, the eye tissue level of PEDF was dampened in db/db mice (Figure 2F). In contrast, oral administration of 10 mg/kg of chrysin restored PEDF to the level of db/m control mice.

### 3.3. Protective Effects of Chrysin on RPE65 Induction 

**Figure 3:**
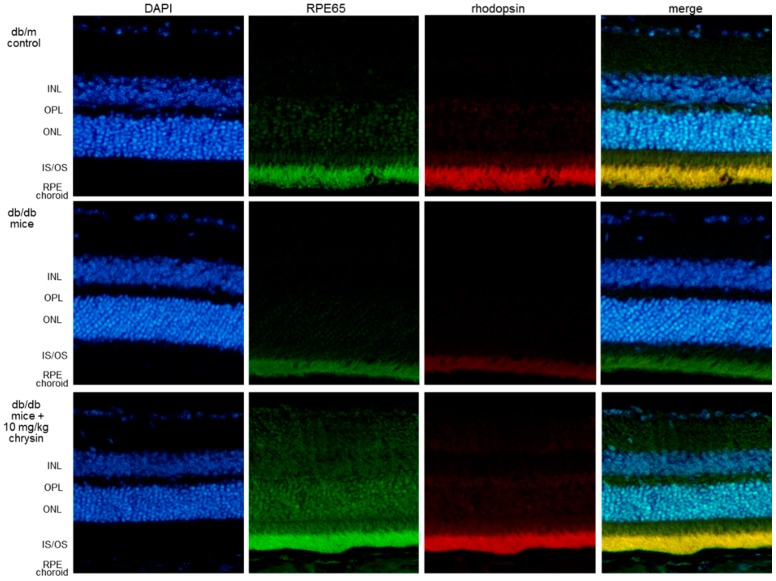
Induction of RPE65 and rhodopsin by chrysin. The db/db mice were orally supplemented with 10 mg/kg of chrysin daily for 10 weeks. The db/m mice were introduced as control animals. Histological sections of mouse retina were immunohistochemically double-stained using a primary antibody of RPE5 and rhodopsin. The RPE65 was identified as fluorescein isothiocyanate (FITC) green staining, and the rhodopsin localization was detected with Cy3 red staining. The sections were counterstained with 4′,6-diamidino-2-phenylindole (DAPI, blue) for the nuclear staining. The triple staining of DAPI-FITC-Cy3 was merged. Magnification: 200×. Retinal layers are labeled as follows: inner nuclear layer (INL), outer plexiform layer (OPL), outer nuclear layer (ONL), photoreceptor inner segment/outer segment (IS/OS), and retinal pigment epithelium (RPE).

The visual cycle occurring between the photoreceptors and RPE regenerates 11-cis-retinal through a series of steps involving specialized enzymes and retinoid binding proteins [18,36]. RPE65 is a critical enzyme responsible for the conversion of all-trans-retinyl esters to 11-cis-retinal during phototransduction [17]. When RPE cells were stimulated with 33 mM of glucose, the cellular expression of RPE65 markedly decreased (Figure 2E). However, treatment with ≥1 μM of chrysin elevated the RPE65 expression to the glucose control level. This study attempted to show that chrysin ameliorated the RPE65 induction in diabetic animal retina. As expected, the eye tissue levels of RPE65 and rhodopsin dropped in db/db mice (Figure 2F). When db/db mice were orally administrated with 10 mg/kg of chrysin for 10 weeks, the tissue levels of these proteins were boosted. Also, the induction of RPE65 and rhodopsin was examined in mouse retina by using a double immunohistochemical staining of FITC and Cy3. In db/m control retina, the RPE65 in the photoreceptor inner/outer segment (IS/OS) layer was strongly green-stained, and the retinal rhodopsin was red-stained in retina (Figure 3). However, there was a weak staining of RPE and rhodopsin in the IS/OS layer of diabetic mouse retina, as compared to that of the db/m control. Oral supplementation of chrysin to db/db mice improved the induction of RPE65 and rhodopsin, in which the double staining of FITC and Cy3 was indistinguishable from that of the db/m control (Figure 3).

### 3.4. Protective Effects of Chrysin on Induction of Visual Cycle-Related Proteins

**Figure 4:**
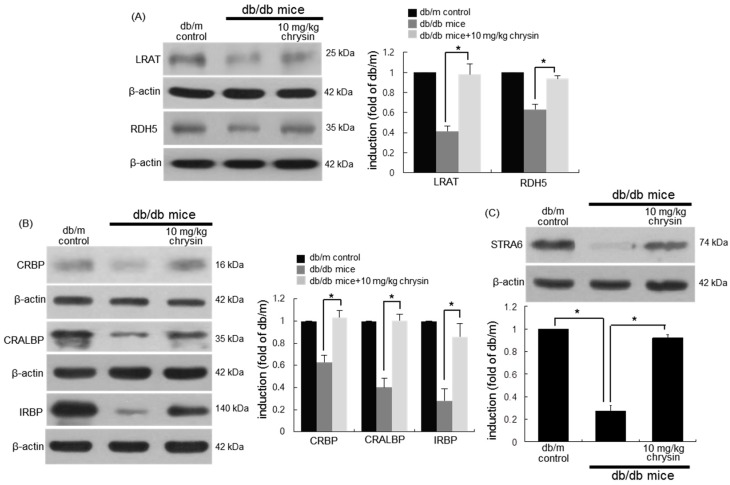
Elevation of retinal tissue induction of visual cycle enzymes (**A**), and retinoid binding proteins (**B**), and stimulated by retinoic acid 6 (STRA6, **C**) in chrysin-treated mice. The db/db mice were orally supplemented with 10 mg/kg of chrysin daily for 10 weeks. The db/m mice were introduced as control animals. Mouse retinal tissue extracts were subject to Western blot analysis with a primary antibody against each target protein of lecithin-retinol acyltransferase (LRAT), retinol dehydrogenase 5 (RDH5), cellular retinol binding protein (CRBP), cellular retinaldehyde-binding protein (CRALBP), interphotoreceptor retinoid-binding protein (IRBP), or STRA6. β-actin protein was used as an internal control. Bar graphs (mean ± SEM, *n* = nine independent experiments) in the bottom or right panels represent densitometric results of upper blot bands. * Values in bar graphs indicate a significant difference at *p* < 0.05.

This study examined whether the hyperglycemic insult influenced the induction of other visual cycle-related enzymes of LRAT and RDH5, and whether chrysin improved the dysfunction of retinoid visual cycle by glucose. The eye tissue levels of LRAT and RDH5 declined in diabetic mice (Figure 4A), while chrysin increased their levels. In addition, the tissue levels of the retinoid binding proteins of CRBP, CRALBP, and IRBP were diminished in eyes of db/db mice (Figure 4B). In contrast, the levels of these binding proteins were nearly completely restored to those of normal mouse retina. This study further examined the level of the vitamin A receptor of STRA6 in diabetic retina. There was a marked loss of STRA6 observed in diabetic eyes (Figure 4C). In chrysin-treated mouse eyes, the STRA6 level was significantly elevated. Accordingly, chrysin ameliorated the diabetes-associated malfunction of the retinoid visual cycle.

### 3.5. Blockade of AGE-Mediated Malfunction of Visual Cycle by Chrysin

**Figure 5:**
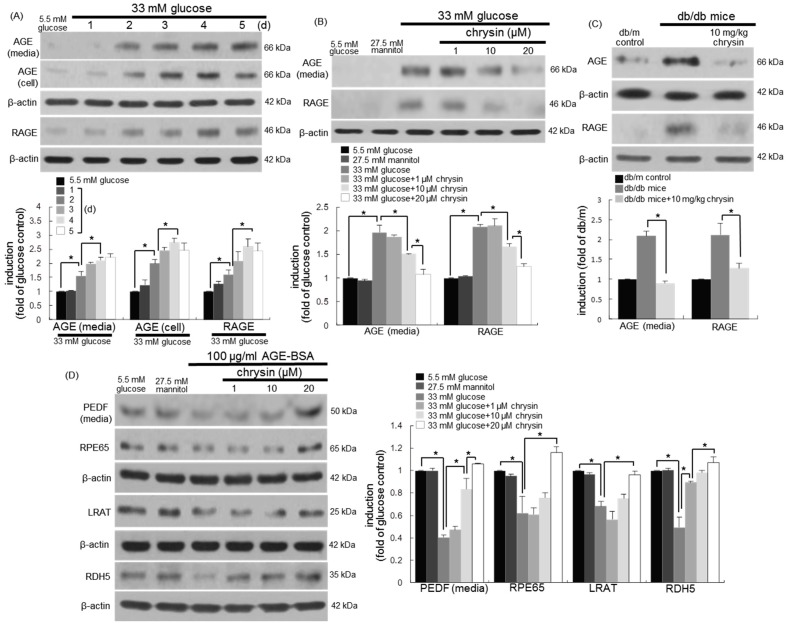
Temporal induction of advanced glycation end products (AGE) and receptor for advanced glycation end product (RAGE) by glucose (**A**), the inhibitory effects of chrysin on their induction (**B**,**C**), and restoration by chrysin of PEDF and retinal visual cycle enzymes in AGE-BSA-exposed human retinal pigment epithelial (RPE) cells (**D**). RPE cells were incubated with 33 mM of glucose in the absence and presence of 1–20 μM chrysin for up to five days. Cells were also incubated with 5.5 mM of glucose and 27.5 mM of mannitol as osmotic controls. The db/db mice were orally supplemented with 10 mg/kg of chrysin daily for 10 weeks. The db/m mice were introduced as control animals. Culture media, cell lysates, and mouse retinal tissue extracts were subject to Western blot analysis with a primary antibody against AGEs, RAGE, PEDF, RPE65, lecithin retinol acyltransferase (LRAT), and retinol dehydrogenase 5 (RDH5). β-actin protein was used as an internal control. Bar graphs (mean ± SEM, *n* = nine independent experiments) in the bottom or right panel represent densitometric results of upper or left blot bands. * Values in bar graphs indicate a significant difference at *p* < 0.05.

High glucose prompted the AGE production and RAGE induction in RPE cells in a time course-dependent manner for six days (Figure 5A). The cellular induction of AGEs and RAGE was highly enhanced on two days post-stimulation with 33 mM of glucose. However, RPE cells exposed to glucose in the presence of 1–20 μM chrysin for three days demoted the AGE secretion and RAGE induction (Figure 5B). Consistently, the tissue levels of AGEs and RAGE were enhanced in diabetic eyes (Figure 5C). In contrast, their levels were diminished in diabetic mice orally treated with 10 mg/kg of chrysin for 10 weeks (Figure 5C).

To investigate the involvement of AGEs in the glucose-induced malfunction of the visual cycle, 100 μg/mL of AGE-BSA were treated to RPEC for three days in the absence and presence of 1–20 μM chrysin. The PEDF secretion declined in AGE-BSA-treated RPE cells (Figure 5D). In addition, the induction of visual cycle-related proteins of RPE 65, LRAT, and RDH5 decreased in AGE-exposed RPE cells. In contrast, the presence of 20 μM of chrysin elevated the induction of these proteins dampened by 100 μg/mL of AGE-BSA (Figure 5D). Therefore, AGEs produced from RPE cells may be responsible for the glucose-induced loss of visual cycle-related proteins.

### 3.6. Involvement of ER Stress in Loss of Visual Cycle Proteins

**Figure 6:**
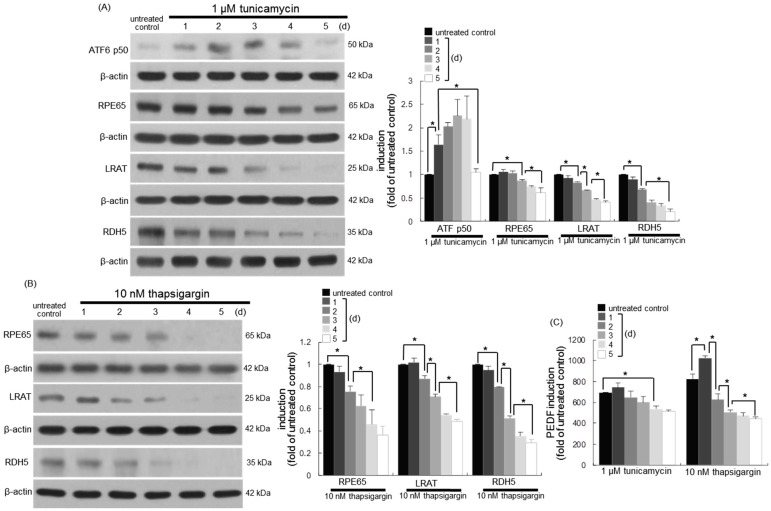
Temporal inhibition of retinal visual cycle enzymes (**A**,**B**) and PEDF (**C**) in endoplasmic reticulum (ER) stress-faced human retinal pigment epithelial (RPE) cells. RPE cells were incubated in the absence and presence of 1 μM of tunicamycin and 10 nM of thapsigargin for up to five days. Cell lysates were subject to Western blot analysis with a primary antibody against activating transcription factor 6 (ATF6) p50, RPE65, LRAT, and RDH5 (**A**,**B**). β-actin protein was used as an internal control. Bar graphs (mean ± SEM, *n* = nine independent experiments) in the right represent the densitometric results of left blot bands (**A**,**B**). The secretion of PEDF in RPE cell culture media was measured with an ELISA kit (**C**). * Values in bar graphs indicate a significant difference at *p* < 0.05.

Aberrant functions take place in ER by glucose insult, which promotes the apoptotic demise of retinal cells in diabetic eyes and impairs visual function [6,7]. This study examined whether ER stress influenced the induction of the retinoid visual cycle enzymes of RPE65, LRAT, and RDH5 located on the smooth ER of RPE cells. As expected, the ER stress inducer tunicamycin resulted in marked ATF6 activation on the day two in RPE cells cultured for up to five days (Figure 6A). During ER stress, the gradual loss of RPE65, LRAT, and RDH5 temporally occurred in tunicamycin-exposed RPEC (Figure 6A). Marked reduction of these proteins was observed at three days-post stimulation. In addition, another ER stress inducer thapsigargin, which is a specific inhibitor of the sarcoplasmic/endoplasmic reticulum Ca^2+^-ATPase, caused the gradual reduction of the visual cycle enzymes of RPE65, LRAT, and RDH5 in RPE cells for five days (Figure 6B). These proteins were near-completely lost at three days-post stimulation with 10 nM of thapsigargin. Furthermore, the PEDF secretion was diminished in RPE cells stimulated with both tunicamycin and thapsigargin for up to five days (Figure 6C).

### 3.7. Inhibition of ER Stress-Mediated Loss of Visual Cycle Proteins by Chrysin

**Figure 7:**
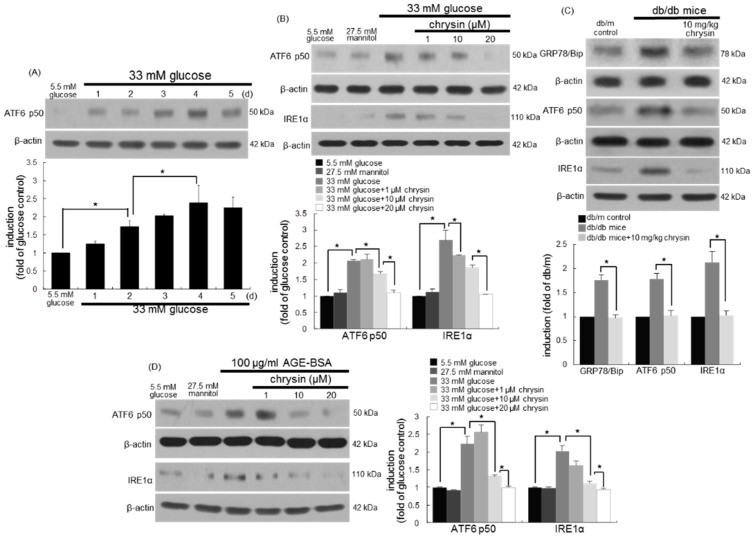
Induction of ER stress by glucose and AGE-BSA and its inhibition by chrysin. Human retinal pigment epithelial (RPE) cells were incubated with 33 mM of glucose (**A**,**B**) or 100 μg/mL of AGE-BSA (**D**) in the absence and presence of between 1–20 μM of chrysin for up to five days. Cells were also incubated with 5.5 mM of glucose and 27.5 mM of mannitol as osmotic controls. The db/db mice were orally supplemented with 10 mg/kg of chrysin daily for 10 weeks (**C**). The db/m mice were introduced as control animals. Cell lysates and tissue extracts were subject to Western blot analysis with a primary antibody against ATF6 p50, inositol-requiring enzyme 1α (IRE1α), and 78 kDa glucose-regulated protein/binding immunoglobulin protein (GRP78/Bip). β-actin protein was used as an internal control. Bar graphs (mean ± SEM, *n* = nine independent experiments) in the bottom represent the densitometric results of upper blot or left bands (**A**,**B**). * Values in bar graphs indicate a significant difference at *p* < 0.05.

This study investigated whether glucose and AGEs induced ER stress just as tunicamycin and thapsigargin did. High glucose activated ATF6 in RPE cells from one day-post stimulation (Figure 7A). Another ER stress sensor protein of IRE1α was induced in 33 mM of glucose-exposed RPE cells (Figure 7B). When 1–20 μM chrysin was treated to glucose-stimulated RPE cells, the ATF6 activation and IRE1α induction were dose-dependently attenuated (Figure 7B). Accordingly, chrysin may improve visual function through suppressing the ER stress of RPE cells in diabetic eyes. In fact, the ER stress-related sensors and chaperone of GRP78/Bip, ATF6, and IRE1α were clearly detected in diabetic eyes (Figure 7C). The eye tissue levels of these proteins were dampened in db/db mice orally administrated with 10 mg/kg of chrysin for 10 weeks. Finally, 100 μg/mL of AGE-BSA per se prompted both ATF6 activation and IRE1α induction in RPE cells, evoking ER stress (Figure 7D). However, the chrysin treatment of glucose-stimulated RPE cells reduced the induction and activation of ER stress-related sensors. Collectively, chrysin may ameliorate the malfunction of the retinoid visual cycle in diabetic retina through combating AGE-induced ER stress.

## 4. Discussion

**Figure 8:**
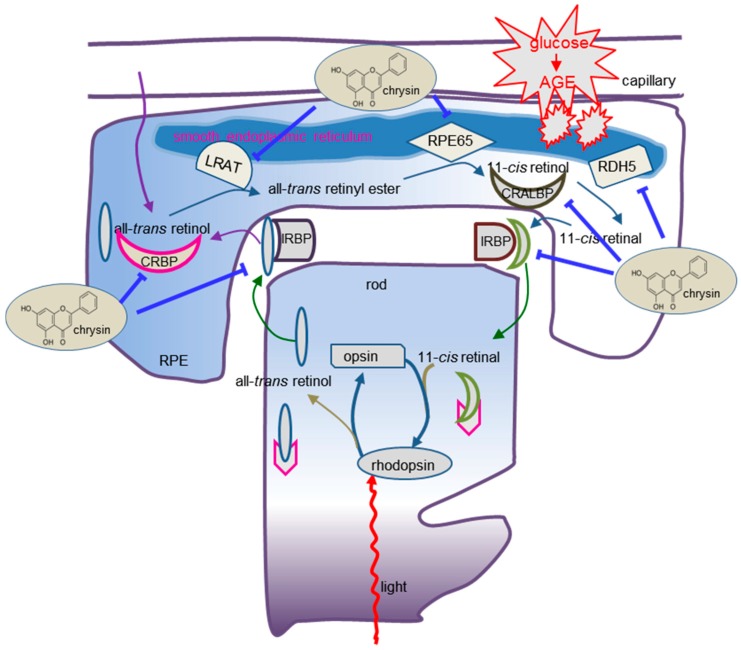
Schematic diagram showing the inhibitory effects of chrysin on the malfunction of retinoid visual cycle and its mechanistic actions in glucose/AGE-exposed human retinal pigment epithelial (RPE) cells. Chrysin inhibited the ER stress activated by glucose/AGEs. All of the arrows indicate increase, activation or induction; ⊥ indicates inhibition or blockade.

Ten major findings were extracted from this study. (1) Glucose influenced the production of VEGF, IGF-1, and PEDF in RPE cells, which was reversed by treating chrysin. (2) The ONL thickness of chrysin-administrated retina was enhanced. (3) The RPE65 reduction in glucose-exposed RPE cells was enhanced by chrysin. (4) Oral administration of chrysin for 10 weeks augmented the protein levels of RPE65, and diminished rhodopsin in db/db mouse retina. (5) The reduced eye tissue levels of LRAT and RDH5 increased in chrysin-administrated diabetic mice. (6) The diabetic tissue levels of CRBP, CRALBP, IRBP, and STRA6 were restored to those of normal mouse retina. (7) The presence of chrysin demoted the AGE secretion and RAGE induction in RPE cells exposed to glucose. (8) Supplementing chrysin to diabetic mice diminished the eye tissue levels of AGEs and RAGE. (9) The reduced induction of PEDF, RPE65, LRAT, and RDH5 was elevated by chrysin in AGE-BSA-exposed RPE cells. (10) The treatment of RPE cells with chrysin reduced the activation of ER stress. These results indicated that chrysin may ameliorate the malfunction of retinoid visual cycle in diabetic retina through combating AGE-induced ER stress (Figure 8).

There is increasing evidence that angiogenic growth factors and cytokines, including VEGF and IGF-1, play crucial roles in proliferative DR development [37]. The intraocular neovascularization is counteracted by the formation of anti-angiogenic factors such as PEDF and transforming growth factor-β (TGF-β). These factors could be used as markers for disease prognosis and therapy. However, several inhibitors of angiogenic factors, including VEGF and placental growth factor, have failed to demonstrate significant efficacy in the treatment of DR in clinical trials [38]. Natural compounds with minimal side effects for intraocular angiogenesis may serve as specific and efficacious agents for a potential DR therapy [39]. This study showed that chrysin inhibited the induction of angiogenic VEGF in glucose-stimulated RPE cells, while the induction of antiangiogenic PEDF was up-regulated. Accordingly, the molecules that shift the balance toward PEDF and away from VEGF may prove useful tools in retinal neovascularization. Interestingly, this study showed that glucose temporally attenuated the induction of angiogenic IGF-1 of RPE cells, which was reversed by treating chrysin. A recent study shows that IGF-1 protects RPE cells from amiodarone-mediated injury via activation of PI3K/Akt signaling [40]. In this study, IGF-1 had potential as a protective agent for deterring the AGE-mediated toxicity of glucose.

This study identified protective mechanisms of chrysin that are germane to visual cycle malfunction in RPE. Pharmacotherapy with visual cycle modulators, including oral retinoids, may improve visual acuity and visual fields in blinding diseases that lack effective treatment options [41]. These modulators may show the side effects and lack of proof of efficacy in humans. Chrysin improved the dysfunction of visual cycle by glucose through enhancing the reduced expression of the rod visual cycle enzymes of RPE65, LRAT, and RDH5 localized on the smooth ER of RPE cells. This study further found that chrysin boosted the induction of all of the retinoid binding proteins of CRALBP and CRBP in RPE cells and IRBP in the subretinal space that diminished in diabetic retina. Unfortunately, this study did not examine the accumulation of all-trans-retinol and all-trans-retinyl esters in RPE cells due to a lack of LRAT, RPE65, and RDH5 enzymes, and did not measure the tissue level of the visual chromophore of 11-cis-retinal, which is the light-sensitive component of visual pigments. Nevertheless, chrysin could facilitate the efficient retinoid cycling between the rod outer segment and the RPE through maintaining optimal levels of visual cycle enzymes in RPE cells, as well as retinoid binding proteins. Several studies have demonstrated that dietary flavonoids attenuate retinal degeneration through deterring the apoptosis of RPE cells and photoreceptors via the inhibition of retinal oxidative stress and inflammation [42,43,44]. However, little is known about the protective roles of dietary compounds in the alteration of rod visual cycle components. This is the first study demonstrating the protective effects of chrysin on the malfunction of visual cycle components that are present in diabetic RPE and photoreceptors.

The molecular mechanisms underlying the inflammatory pathways associated with DR are not concretely defined. In addition, a mutual connection between oxidative stress and major metabolic abnormalities has been implicated in the development of DR [45,46]. Curcumin inhibits oxidative stress and protects Müller cells in diabetic retina [39,47]. Recently, there has been much advance study in the possible molecular mechanisms leading to autophagy that are involved in the pathophysiology of DR [48]. On the other hand, the AGE-RAGE system plays a crucial role in eliciting oxidative stress and inflammatory reactions, and is involved in diabetic damage [49]. This study revealed that the AGE-RAGE system was activated in glucose-stimulated RPE cells and diabetic eyes, ultimately inducing visual cycle dysfunction in retina. Chrysin diminished the AGE accumulation and RAGE induction in glucose-exposed RPE cells and diabetic eyes. Various inhibitors of the AGE-RAGE system have been evaluated as their therapeutic utility for DR [49]. The antioxidant sulforaphane that is present in edible cruciferous vegetables inhibits AGE-induced pericyte injury through blocking the AGE-RAGE axis in pericytes, which is a novel therapeutic target for the treatment of DR [50].

Numerous studies have addressed that ER stress plays a putative role in the visual function [6,9,51]. Oxidative stress and ER stress contribute to the progression of age-related macular degeneration, which is characterized by retinal degeneration resulting in the loss of central vision [52]. In this study, AGEs promoted the induction of ER stress sensor proteins of ATF6 and IRE1α in RPE cells. In addition, the ER stress inducers of tunicamycin and thapsigargin dampened the expression of visual cycle components in retinal pigment epithelium. These results identified ER stress as a negative regulator of the RPE visual cycle. Since the dysfunction of any retinoid cycle enzymes in the RPE can cause ocular diseases, the blockade of glucose-triggered ER stress by chrysin may be a manipulative strategy to protect retinal degeneration and the vision loss of DR. Moreover, ER stress is a critical adverse component of RPE cells and photoreceptors [53,54]. One investigation shows that the tea polyphenol (−)epigallocatechin gallate inhibited ER stress-mediated apoptotic cell death via the proper calcium homeostasis and decreased ROS production in age-related macular degeneration [55]. Bilberry extract attenuates photoinduced apoptosis and visual dysfunction via ER stress attenuation in the retina [54]. The accumulation of misfolded rhodopsin within the ER is a prominent cause of retinitis pigmentosa [56]. This study found that the tissue level of rhodopsin declined in diabetic eyes, which was ameliorated by implementing the chrysin strategy. The chrysin supplementation could enhance the clearance of misfolded rhodopsin and maintain proper unfolded protein response signaling in the ER.

In summary, this study investigated the capability of chrysin in counteracting the diabetes-mediated malfunction of the visual cycle in glucose-exposed RPE cells and diabetic mice. Chrysin reciprocally influenced the production of VEGF, IGF-1, and PEDF in glucose-stimulated RPE cells and diabetic eyes. This compound neutralized the hyperglycemia-elicited reduction of the retinoid visual cycle components of RPE65, LRAT, RDH5, CRBP, CRALBP, IRBP, and STRA6 in retina. In addition, chrysin encumbered the formation of AGEs and RAGE in diabetic eyes due to glucose insults, leading to the loss of RPE enzymes for the visual cycle. Furthermore, chrysin blocked the ER stress of RPE cells evoked by glucose and AGEs in diabetic mice. Therefore, chrysin was a therapeutic drug antagonizing the malfunction of the retinal visual cycle, leading to the loss of retinal vision in cellular or animal models of diabetic complications. It can be assumed that the protective effects of chrysin on the malfunctioned visual cycle in diabetic eyes may be, at least partly, due to its glucose-lowering effects.
